# Effects of Enhanced Hydrophilic Titanium Dioxide-Coated Hydroxyapatite on Bone Regeneration in Rabbit Calvarial Defects

**DOI:** 10.3390/ijms19113640

**Published:** 2018-11-19

**Authors:** Ji-Eun Lee, Chung Wung Bark, Hoang Van Quy, Seung-Jun Seo, Jae-Hong Lim, Sung-A Kang, Youngkyun Lee, Jae-Mok Lee, Jo-Young Suh, Yong-Gun Kim

**Affiliations:** 1 Department of Periodontology, School of Dentistry, Kyungpook National University, Daegu 41940, Korea; lufinel@naver.com (J.-E.L.); juns@knu.ac.kr (S.-J.S.); leejm@knu.ac.kr (J.-M.L.); jysuh@knu.ac.kr (J.-Y.S.); 2 Department of Electrical Engineering, Gachon University, Seongnam 13120, Korea; bark@gachon.ac.kr (C.W.B.); quybk20113989@gmail.com (H.V.Q.); 3 Industrial Technology Convergence Center, Pohang Accelerator Laboratory, Pohang University of Science and Technology (POSTECH), Pohang, Gyeongbuk 37673, Korea; limjh@postech.ac.kr; 4 Advanced Bio Convergence Center, Pohang Technopark, Pohang 37668, Korea; kang5239@ptp.or.kr; 5 Department of Biochemistry, School of Dentistry, Kyungpook National University, Daegu 41940, Korea; ylee@knu.ac.kr

**Keywords:** regeneration, calvarial defect, photofunctionalization, ultraviolet, titanium dioxide, hydrophilicity

## Abstract

The regeneration of bone defects caused by periodontal disease or trauma is an important goal. Porous hydroxyapatite (HA) is an osteoconductive graft material. However, the hydrophobic properties of HA can be a disadvantage in the initial healing process. HA can be coated with TiO_2_ to improve its hydrophilicity, and ultraviolet irradiation (UV) can further increase the hydrophilicity by photofunctionalization. This study was designed to evaluate the effect of 5% TiO_2_-coated HA on rabbit calvarial defects and compare it with that of photofunctionalization on new bone in the early stage. The following four study groups were established, negative control, HA, TiO_2_-coated HA, and TiO_2_-coated HA with UV. The animals were sacrificed and the defects were assessed by radiography as well as histologic and histomorphometric analyses. At 2 and 8 weeks postoperatively, the TiO_2_-coated HA with UV group and TiO_2_-coated HA group showed significantly higher percentages of new bone than the control group (*p* < 0.05). UV irradiation increased the extent of new bone formation, and there was a significant difference between the TiO_2_-coated HA group and TiO_2_-coated HA with UV group. The combination of TiO_2_/HA and UV irradiation in bone regeneration appears to induce a favorable response.

## 1. Introduction

The regeneration of bone defects caused by infection or trauma is an important goal in clinical practice, and tissue engineering has been used to control the process of wound healing so that the tissue can be regenerated. The three crucial factors in tissue engineering are cells, scaffold (supporting matrices), and signaling molecules. In terms of the scaffold, hydroxyapatite (HA) is a bone material with osteoconductivity [1]. The porous structure of HA promotes the ingrowth of cells and microvessel growth leading to bone formation. Moreover, for this bone material, optimal pore sizes from 150 to 500 μm are an important factor in forming new bone [2].

Even though the adhesion of osteoblast cells to the surface of a scaffold is a crucial factor for the healing of hard tissue, many materials, such as HA or titanium, have a disadvantage in the initial healing process because of their hydrophilicity. To solve this problem, previously, TiO_2_ and UV treatments of the surface of materials were adopted. For example, there is biocompatibility based on the formation of a thin TiO_2_ layer [3]. It has been reported that treatment of the surface of dental implants with TiO_2_ improves cell adhesion and osseointegration. Fujibayashi and his colleagues [4] reported in their study on porous titanium material that the complex interconnective macroporous structure improves osteoconductivity. However, titanium and these alloys have been reported to have disadvantages such as low tissue adherence and a degree of toxicity caused by release during corrosion [5,6]. Niska and her colleagues [7] demonstrated that toxicity induced by TiO_2_ nanoparticles cause oxidative damage by increasing levels of O_2_^•−^ (superoxide anion) in human osteoblast cells. On the other hand, Mani et al. [8] reported in their in vivo study that hydroxyapatite attenuates the toxicity of TiO_2_. In addition, Okumura et al. [9] studied the initial attachment of osteoblast cells (Saos-2) to different materials, and they reported superior early adhesion of human osteoblast-like cells to HA and titanium than to other materials. Therefore, a combination of HA and TiO_2_ for bone regeneration is considered appropriate.

According to several reports on how the surface properties of dental implants improve osseointegration, titanium and these alloys do not show reliable bone-to-implant contact [4,10]. They concluded that modifying surface characteristics is necessary to improve osseointegration. Zhao and his colleagues [11] reported in their study that osteoblasts cultured on hydrophilic surfaces express higher levels of differentiation markers, such as alkaline phosphatase and osteocalcin and generated an osteogenic microenvironment. Ultraviolet (UV) photofunctionalization has been widely studied as a means to increase hydrophilicity to further improve the effect of a scaffold on bone formation [12,13,14]. Radiating the titanium surface with UV enhances the physical properties. One review reported that the unique alteration of the titanium surface after UV treatment is attributed to the surface of TiO_2_, which is changed to hydrophilic at the atomic level [13]. More specifically, UV irradiation of the titanium surface converts Ti^4+^ into Ti^3+^, which is beneficial for adsorption and makes the titanium surface bioactive by becoming hydrophilic. Thus, in several studies, UV photofunctionalization has been reported as a method for increasing cell attachment and new bone formation to this more hydrophilic surface [12,15,16]. The use of scaffold carriers with increased hydrophilicity is expected to further enhance the initial healing response for regeneration, including angiogenesis and osseointegration.

To reduce nanotoxicity due to nanosized TiO_2_ particles, we have attempted to lower the ratio of TiO_2_ by adding a surfactant. An in vitro pilot study indicated that a rapid change in terms of hydrophilicity occurs when concentration of nanosized TiO_2_ is varied from 1.5 to 5%, as indicated by the change in the contact angle. Therefore, in this study, UV photofunctionalization was examined in 5% TiO_2_ to determine the minimum dose. This experiment was designed to evaluate the effect of using HA with 5% TiO_2_ on rabbit calvarial defect models as a bone graft material and to perform histologic and histomorphometric comparisons of the effect of ultraviolet photofunctionalization on new bone formation in the early phase of the healing response.

## 2. Results

### 2.1. Surface Characteristics

**Figure 1:**
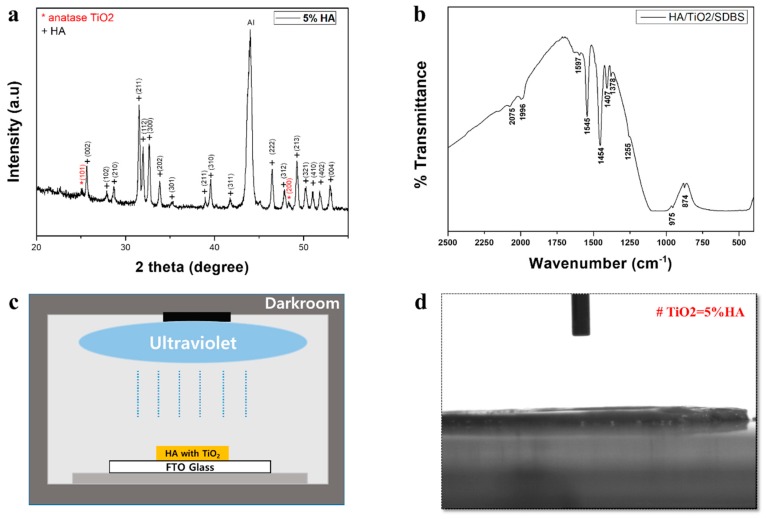
(**a**) X-ray diffraction spectroscopy (XRD) analysis. Anatase TiO_2_ is present in TiO_2_-coated HA. (**b**) Fourier transform infrared spectroscopy (FTIR) measurement to check the biocompatibility of the combined graft material. (**c**) Photofunctionalization of TiO_2_-coated HA particles by irradiating with ultraviolet light for 1 min using a photo device. (**d**) Wettability test. For the 5% TiO_2_ sample in the mixture, the contact angle was maintained at 0°.

Figure 1a shows the XRD pattern of the HA/TiO_2_ sample. The XRD analysis indicated that TiO_2_ adheres well to the HA surface. The low intensity of the TiO_2_ peak was thought to be due to the minimum concentration of TiO_2_ present in the mixture. The XRD peaks clearly indicate the HA and TiO_2_ phases and suggest that a mutual reaction between HA and TiO_2_ and decomposition did not occur because the calcination temperature was kept at only 450 °C. A sodium dodecylbenzenesulfonate (SDBS) peak was not observed because it had already been removed during the thermal annealing. Figure 1b shows the FTIR spectrum of the HA/TiO_2_ sample. The chemical groups of the HA particles in the FTIR spectrum are ${\mathrm{PO}}_{4}^{3-}$, ${\mathrm{OH}}^{-},$ and ${\mathrm{CO}}_{3}^{2-}$. The FTIR spectrum of the coatings clearly illustrates the low presence of both titania phases (400–800 cm^−1^) and the presence of HA. The FTIR analysis indicated that SDBS did not change the chemical composition of the HA/TiO_2_ sample. The spectrum for SDBS did not appear, agreeing with the XRD measurement. The FTIR measurement confirmed that SDBS was volatilized after the sintering process. Using UV equipment and geometry (Figure 1c), UV light was used to irradiate the HA/TiO_2_ sample. Figure 1d presents the contact angle results of HA/TiO_2_ coating after UV exposure. The sample showed superior wettability compared with the HA cases. The surface-prepared sample was easily wetted, resulting in a sharp decrease in the contact angle to nearly 0°.

### 2.2. Histology

All animals survived the surgical procedure, and the clinical healing process was uneventful, without postoperative complications such as surgical wound dehiscence, signs of inflammation, edema, or other complications. Six rabbits were sacrificed two weeks after surgery, and the remaining six rabbits were sacrificed after eight weeks.

**Figure 2:**
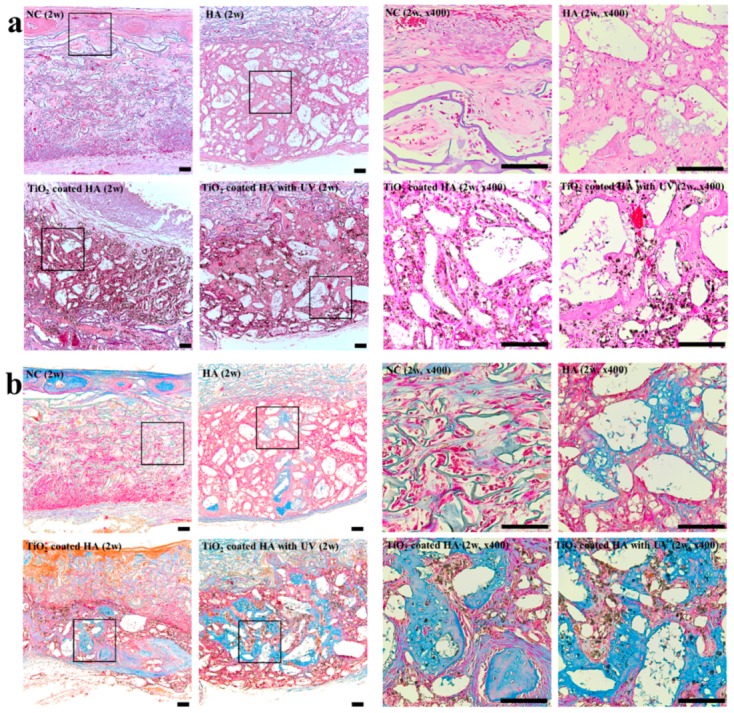
Histological findings (2 weeks). (**a**) **Left**: Light microscopy using hematoxylin and eosin (H&E) stain (×100) showing new bone formation in the groups at two weeks postoperatively; NC group, HA group, TiO_2_-coated HA group, and TiO_2_-coated HA with UV group. In UV-treated group (lower-left), there was significantly more newly formed bone than in the other groups. **Right**: Magnification of (**a**) (×400). Scale bar: 100 um; (**b**) **Left**: Light microscopy using Masson trichrome stain (×100) showing new bone formation in the groups at two weeks postoperatively; NC group, HA group, TiO_2_-coated HA group, and TiO_2_-coated HA with UV group. In the UV-treated group (lower-left), the residual graft materials were surrounded by blue-colored woven bone. **Right**: Magnification of (**b**) (×400). Scale bar: 100 um.

At two weeks, most of newly formed bone was woven bone with large numbers of osteoclasts, as shown in Figure 2. Bone regeneration appeared to extend from the border of the surgical defect to the center. TiO_2_ was observed as a black-colored particle in the TiO_2_-coated HA group and TiO_2_-coated HA with UV group. In contrast to the negative control group, in which little newly formed bone was observed, woven bone formed around the bone graft materials in the three other groups (Figure 2a). In Masson trichrome staining, the immature woven bone was stained blue, and abundant and dense immature bone was clearly observed in the TiO_2_-coated HA with UV group (Figure 2b).

**Figure 3:**
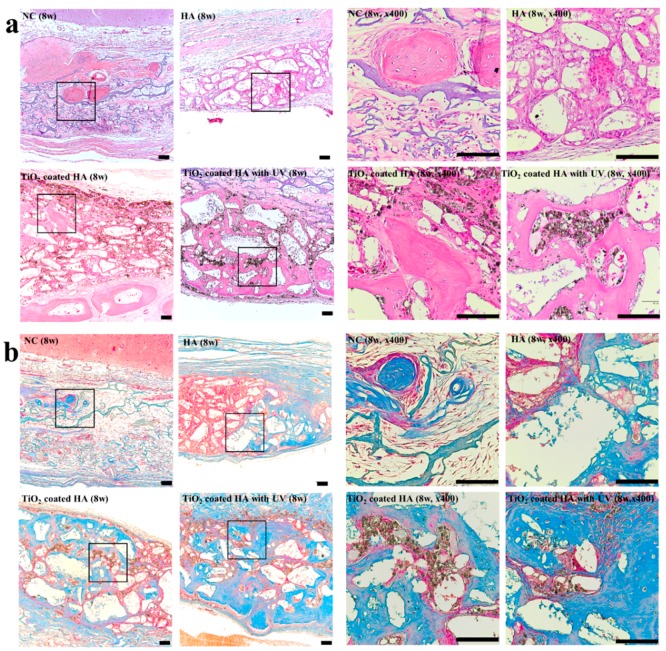
Histological findings (eight weeks). (**a**) **Left**: Light microscopy using H&E stain (×100) showing new bone formation in the groups at eight weeks postoperatively; NC group, HA group, TiO_2_-coated HA group, and TiO_2_-coated HA with UV group. The amount of new bone formation in all groups was greater than that at 2 weeks after surgery. New bone formation in the TiO_2_-coated HA with UV group was more distinct than that in the other groups. **Right**: Magnification of (**a**) (×400). Scale bar: 100 um. (**b**) **Left**: Light microscopy using Masson trichrome stain (×100) showing new bone formation in the groups at 8 weeks postoperatively; NC group, HA group, TiO_2_-coated HA group, and TiO_2_-coated HA with UV group. UV-treated group (lower-left). **Right**: Magnification of (**b**) (×400). Scale bar: 100 um.

Histologic analysis of new bone formation at 8 weeks (Figure 3a,b) revealed that the amounts of new bone in the surgical defect increased in all groups compared with that at two weeks. We also observed more mature bone with moderate amounts of the bone marrow. At eight weeks, most of the defects in the TiO_2_-coated HA with UV group were filled with new bone.

### 2.3. Histomorphometry

**Figure 4:**
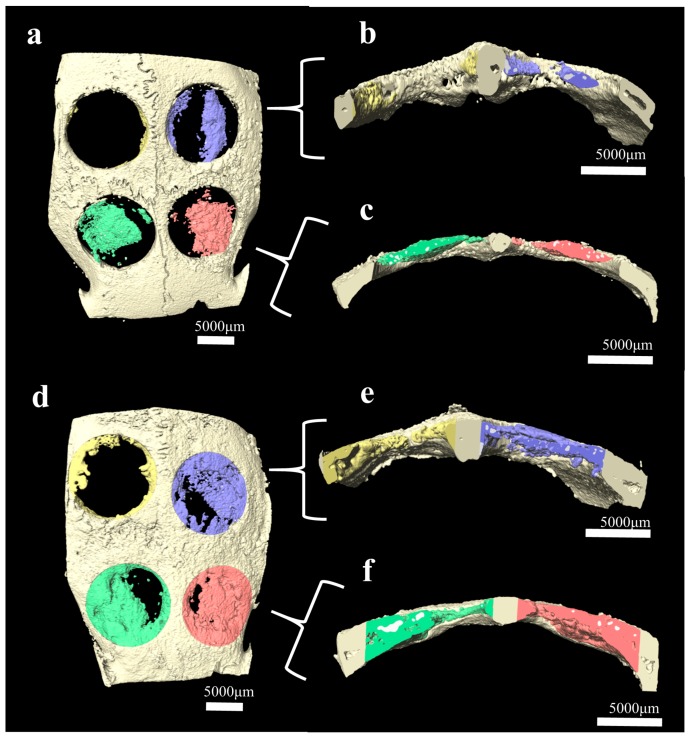
Comparison of regenerated new bone volume at 2 weeks (**a**–**c**) and eight weeks (**d**–**f**) after surgery. The defects from the four groups are represented in different colors as follows: the NC group (yellow), HA group (blue), TiO_2_-coated HA group (green), and TiO_2_-coated HA with UV group (red). 3D visualization of a rabbit calvarial defect specimen acquired by micro-CT for (**a**,**d**) a whole specimen containing new bone and substitutes, (**b**,**e**) cross-section image of the NC group and HA group, and (**c**,**f**) cross-section image of the TiO_2_-coated HA group and TiO_2_-coated HA with UV group.

The measured value of the degree of new bone formation gradually increased over time. As shown in Figure 4 and Table 1, there was a significant difference between two and eight weeks in all four groups. In the evaluation of the residual bone material percentage, only the TiO_2_-coated HA group showed a significant difference between two and eight weeks after the surgery (*p* < 0.05).

**Figure 5:**
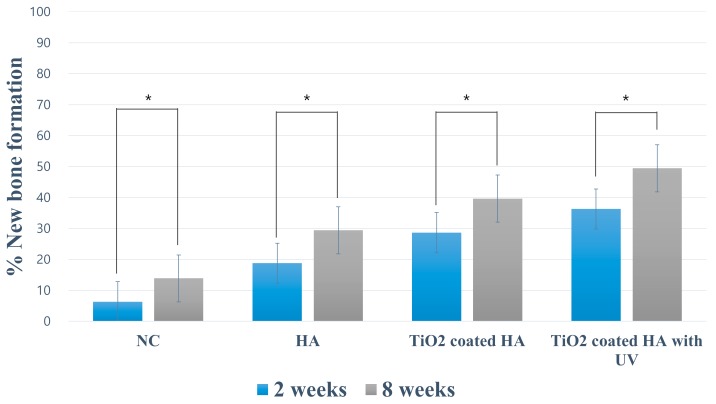
Histomorphometric evaluation of bone regeneration (intragroup analysis). In all groups, statistically significant intragroup differences in new bone formation were observed at two weeks and eight weeks. Data are presented as the mean ± standard error of the mean. * *p* < 0.05.

The percentages of new bone and residual bone material at two and eight weeks postoperatively are listed in Table 1 and Figure 5. These values of histologic and histomorphometric evaluations were done directly from the histologic slides. The newly formed bone ratios in the surgically created defect at two weeks after the surgery were 6.36 ± 1.72% (NC group), 18.78 ± 4.60% (HA group), 28.63 ± 2.75 % (TiO_2_-coated HA group), and 36.32 ± 6.87 % (TiO_2_-coated HA with UV group), and the percentages of residual bone material were 10.56 ± 1.62% (HA group), 10.90 ± 1.78% (TiO_2_-coated HA group), and 14.53 ± 2.15 % (TiO_2_-coated HA with UV group). The TiO_2_-coated HA with UV group had a significantly higher value of new bone formation than the three other groups, and there was a significant difference between the groups (*p* < 0.05).

The newly formed bone ratio was 13.90 ± 3.68% (NC group), 29.42 ± 5.81% (HA group), 39.68 ± 6.70% (TiO_2_-coated HA group), and 49.46 ± 6.54% (TiO_2_-coated HA with UV group) at eight weeks postoperatively, and the TiO_2_-coated HA with UV group had a significantly higher new bone percentage than the three other groups (*p* < 0.05). The TiO_2_-coated HA group had significantly higher new bone formation than the NC and HA groups (*p* < 0.05). The remaining graft material ratios at 8 weeks were 12.14 ± 5.22% (HA group), 14.64 ± 2.82% (TiO_2_-coated HA group), and 13.38 ± 3.44% (TiO_2_-coated HA with UV group), and no difference between the groups was observed.

## 3. Discussion

The aim of this study was to compare the histological and histomorphometric evaluations of the degree of new bone formation in rabbit calvarial defects when applying HA with 5% TiO_2_ and ultraviolet irradiation to induce bone formation. As mentioned above, the contact angle for the hydrophilicity evaluation changes according to the TiO_2_ concentration in HA, and hence a concentration of 5% TiO_2_ in HA was used in with or without the UV irradiation condition. Moreover, the defect size was 8 mm, which is the critical size requiring additional treatment because the defect will not heal naturally during the life of the animal. Histologic evaluations at two and eight weeks were planned to examine the initial healing response [17,18].

Following the grafting of HA with 5% TiO_2_, new bone formation was significant at the 2nd and 8th weeks of healing compared to that when only HA was applied. Specifically, the TiO_2_-coated HA group and UV treatment group had significantly more new bone formation at two and eight weeks compared with that in the HA only group. This indicated that the effect of TiO_2_ on new bone formation was stronger in the initial healing reaction. This may support the results of a study that reported that the nanoscale anodized titanium surface affects cell differentiation in the initial healing period, thus accelerating osseointegration and bone boding strength [19].

When new bone formation was compared between the TiO_2_-coated HA with UV group and TiO_2_-coated HA group, the percentage of new bone formation was significantly higher in the UV-irradiated group. The higher level of new bone formation in the UV-treated group was probably due to the electron excitation effect of the UV irradiation on TiO_2_ mentioned above. Shen et al. [14] investigated the effect of UV irradiation on the bone response in the tibial metaphyses and femoral condyle of rabbits in a histological and histomorphometric study. It had been reported that the nanostructure and UV treatment enhance the interfacial strength of titanium and intergranular bone to improve osseointegration. This conclusion is supported by the results of this experiment, despite the fact that different sites were evaluated, although the same experimental animals were used.

For this experiment, UV irradiation was performed for 1 min. In many studies, longer UV exposure times result in a more hydrophilic surface because of photofunctionalization. M. Q. Tran and his colleague [20] reported that when irradiating with UV for 30 min, the transition from a superhydrophobic to superhydrophilic state occurs faster than with TiO_2_ nanoparticle-coated samples. In addition, a superhemophilic pattern was observed in a wettability test on blood, indicating that the UV irradiation had a potential effect on the clinical application [21]. In a study of the photoinduced hydrophilicity effect, the water contact angle was 54.5° for TiO_2_ film without any treatment and 29.3° for 1 min of UV exposure [22]. For irradiation over 10 min, the result was less than 5°, which indicates superhydrophilicity. However, when the initial healing response indicators such as fibrinogen adsorption and platelet adhesion/spreading were evaluated, there was no significant difference between 1 min and 10 min of UV irradiation; thus, it was concluded that further study was needed. Most other studies have reported that longer irradiation times result in a greater hydrophilic effect by photofunctionalization [15,23,24,25]. However, for clinical use, it is not appropriate to subject the patient to long treatment times, and hence we set a time of 1 min for this study. Additional studies on light irradiation methods will be needed to develop more appropriate clinical applications.

In this study, a heating technique was used to prevent TiO_2_ nanoparticles, which were coated homogeneously on the HA surface, from being separated. In other words, this heating technique, involving heating in a furnace to 450 °C, can prevent the separation phenomenon from occurring during the conventional method of mixing TiO_2_ nanoparticles with HA in ethanol solvent and stirring the mixture ultrasonically. Surface hydrophilicity was evaluated by assessing the contact angle and performing wettability tests, and SEM, XRD, and FTIR measurements were used to detect changes in surface morphology. It was confirmed in SEM and XRD analyses that TiO_2_ was adsorbed uniformly and stably on the surface of HA. Moreover, because SDBS was not present in the FTIR analysis, the biocompatibility of the corresponding sample might be guaranteed. Based on these results, this process was considered to be a suitable method for coating TiO_2_ nanopowder on porous HA.

However, the sample size of the TiO_2_-coated HA particles changed during the process of ultrasonic dispersion. The pore size of the commercial porous HA (Bongros; Daewoong Pharmaceutical Co. Ltd., Seongnam, Korea) was 300 μm, but the mixed graft material changed to a powder form during the stirring process; thus, the particle size and pore size may have decreased. In a study that evaluated the amount of new bone formation after applying various particle sizes of HA to temporal bullae in rats, larger HA particles were associated with faster new bone formation rates [26]. This finding explains why the rate of new bone formation was lower in this experiment when using a sample with smaller particle size than the rate reported in our previous study. Chang et al. studied the osteoconductivity of HA samples with various sizes of cylindrical pores, and they reported that active osteoconduction was also observed in HA with 50-μm cylindrical pores [27]. This can explain the osteoconductivity seen in this experiment, in which the pore size might have decreased. However, further studies on how this affected bone regeneration are needed before conclusions regarding the clinical evaluation and usefulness of the material can be made.

## 4. Materials and Methods

### 4.1. Preparation of Bone Graft Materials

Porous HA (Bongros; Daewoong Pharmaceutical Co. Ltd., Seongnam, Korea) was used for this study as a bone graft material. Sodium dodecylbenzenesulfonate (SDBS) was used as a surfactant to enhance the dispersibility of TiO_2_ nanoparticles on the HA surface. First, 0.2 g of SDBS was completely dissolved in 20 mL of deionized water, and 0.01 g of TiO_2_ nanoparticles was added to the solution under continuous stirring at 200 rpm for 20 min. Then, 0.2 g of HA and TiO_2_/SDBS powders, which were obtained from the centrifugation process, were thoroughly stirred into 20 mL ethanol for 20 min. After that, heat treatment at 450°C for 3 h was applied to the mixture (mixing ratio: HA 0.2 g, TiO_2_ 0.01 g) to remove volatile SDBS.

The microscopic surface structures of porous HA and TiO_2_-coated HA were analyzed using scanning electron microscopy (SU8220, Hitachi High Technologies, Tokyo, Japan) following the platinum coating of the samples. The quantitative and qualitative measurements for the structural identification of the graft material compositions were performed using an energy dispersive X-ray spectrometer (X-ManN50 011; HORIBA, Kyoto, Japan).

### 4.2. Wettability Test

To evaluate hydrophilicity, a wettability test was performed to measure the contact angle of water droplets (interval time: 3 min) with HA and a certain amount of TiO_2_ coated on HA. All the procedures were carried out in a class 10 clean room under the conditions of 20°C and 46% humidity.

### 4.3. UV Photofunctionalization

Photofunctionalization was performed by irradiating the HA graft materials containing TiO_2_ with UV light in a dark room for 1 min using a photo device immediately before grafting. Specifically, the UV treatment was performed at room temperature for a fixed distance of 3 cm between the sample (the irradiated area on the sample was 2 × 2 cm^2^) and the UV light source (UV radiation with a peak at 253.7 nm, power: 8 W).

### 4.4. Animal Study

**Figure 6:**
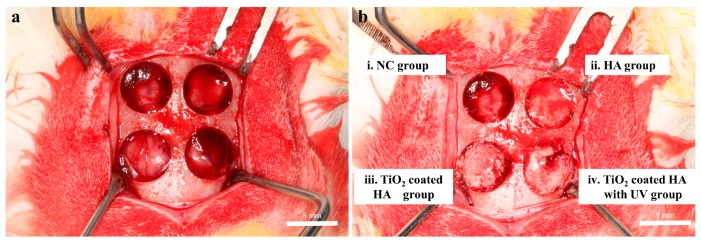
Clinical photographs of the experiment. (**a**) Photograph of four transosseous defects 8 mm in diameter, >2 mm apart, that were created using a trephine bur in the each calvaria of 12 rabbits. (**b**) Defects were filled with the following graft materials. i. Unfilled as a negative control, NC group; ii. 0.025 g of HA, HA group; iii. 0.025 g of TiO_2_-coated HA, TiO_2_-coated HA group; and iv. 0.025 g of TiO_2_-coated HA with UV treatment for 1 min, TiO_2_-coated HA with UV group.

Twelve New Zealand white rabbits weighing 2.7 to 3.2 kg were used in this study. The animals were fed a standard diet and housed in separate cages under standard laboratory conditions. This experiment, including animal selection, management, and the surgery protocol, was approved by the Institutional Animal Care and Use Committee of Kyungpook National University, Daegu, Korea (certification #2017-0094; 05/07/2017). General anesthesia was induced by intramuscular injection of a mixture of zolazepam (0.2 mL/kg; Zoletil, Virbac, Carros, France) and xylazine (0.25 mL/kg; Rompun, Bayer Korea Co., Seoul, Korea). The surgical site was shaved and then covered with alcohol and povidone iodine. Complementary infiltrative anesthesia was administrated at the surgical site using 2% lidocaine with 1/100,000 epinephrine (1:100,000 epinephrine; Yuhan Co., Seoul, Korea) to control bleeding. The surgical site was exposed with a sagittal midline incision through the skin and the periosteum at the midline of the calvaria and a full-thickness flap elevation. Four standardized transosseous circular defects (of critical size: diameter 8 mm) were prepared using a stainless steel trephine bur in the frontal and parietal bones of each animal under cool-saline irrigation (Figure 6a).

Defects were filled with following graft materials (Figure 6b), 0.025 g of HA, HA group; 0.025 g of TiO_2_-coated HA, TiO_2_-coated HA group; 0.025 g of TiO_2_-coated HA, treated with UV for 1 min, TiO_2_-coated HA with UV group; and the last defect was left unfilled as a negative control (NC), NC group. After surgery, surgical defects were covered with a resorbable collagen membrane (collatape; Zimmer, Carlsbad, CA, USA), and the soft tissues were closed in layers and sutured for primary closure using braided polyglycolic acid sutures (5-0 surgifit; AILEE, Busan, Korea) and Monofilament sutures (4-0 nylon; NYLON (blue); AILEE, Busan, Korea). Antibiotics (Baytril, Bayer Korea Co., Seoul, Korea) and analgesics (Nobin, Bayer Korea Co., Seoul, Korea) were injected intramuscularly for 3 days to control pain and prevent postsurgical infection as a postoperative management. Six animals were sacrificed by intravenous injections of air under general anesthesia at 2 weeks postoperatively, and the rest were sacrificed 8 weeks after the surgery. After sacrifice, the area of the surgical defect site and surrounding tissues were removed en bloc. These collected samples were fixed in 4% neutral-buffered paraformaldehyde.

### 4.5. X-ray Microcomputed Tomography Analysis

First, all en bloc samples were subjected to X-ray microtomography. This protocol micro-CT imaging was performed at the Pohang Center for Evaluation of Biomaterials, Pohang Technopark in Pohang, Korea, using a Siemens Inveon Trimodality Image system (INVEON; Siemens, Washington, DC, USA).

The CT slice images were reconstructed using Siemens Inveon Acquisition Workplace Software (INVEON; Siemens, Washington, DC, USA). CT Acquisition: For a whole body CT scan, set current at 500 uA, voltage at 80 kV, exposure time at 280 msec, and 180 steps for 360° rotation. For X-ray detector, select resolution at “low system magnification” with 57.6 mm axial imaging field and single bed mode. We selected “real time reconstruction” using the “Common Cone-Beam Reconstruction” method so that the host PC talks with a dedicated real timereconstruction computer (Cobra) to initiate the task. The corresponding images, produced by stacking all cross-sectional images, create a map of the local X-ray attenuation coefficient plus the enhanced boundaries by the interference of transmitting coherent X-rays. Image analysis software (Amira Version 6.2; FEI Co., Hillsboro, OR, USA) was used for tridimensional (3D) visualization. In the image analysis, newly formed bone fragments were clearly distinguished from the HA in the gray level. Analysis of the 3D images allowed calculating the regenerated total volume of surgical defects and the extent of the newly formed radiopaque area at two weeks and eight weeks postoperatively.

### 4.6. Surface Characteristics and Histological and Histomorphometric Analyses

For histological analysis, the fixed samples were decalcified in 10% ethylenediaminetetraacetic acid (EDTA) for 1 month and then embedded in paraffin. The paraffin-embedded tissues were cut from the center at 3-µm intervals using a Leica microtome (RM2245; Leica Microsystems, Bannockbrun, IL, USA), and the most central sections from each sample were subjected to hematoxylin-eosin (H&E) and Masson’s trichrome staining. Histological evaluations were performed by observing the stained sections under a microscope (Olympus BX53, Olympus, Tokyo, Japan) equipped with a 10 × 0.30 UPLANFL N objective lens (Olympus, Tokyo, Japan). Images were captured using a digital camera (Olympus, Tokyo, Japan) attached to the microscope. Histomorphometric analysis was performed using image analysis software (I-solution; iMTechnology, Daejeon, Korea) to calculate the amount of new bone ingrowth in the defect sites: (1) the percentage of new bone (%) = the area of new bone between the defect margins (mm^2^)/the total augmented area (mm^2^) × 100 (%) and (2) the percentage of residual bone material (%) = the area of residual bone material between the defect margins (mm^2^)/the total augmented area (mm^2^) × 100 (%).

### 4.7. Data Analysis

Statistical analyses were conducted using IBM SPSS Statistics (SPSS Inc., Chicago, IL, USA). Statistical analyses included a nonparametric Kruskal–Wallis analysis of variance test to evaluate differences in the percentages of new bone formation and remaining bone particles. The Mann–Whitney U-test was used to evaluate the difference in bone regeneration between groups. Statistical significance was defined as a *p*-value less than 0.05.

## 5. Conclusions

In this study, it was concluded that the combination of TiO_2_-coated HA and UV treatment for bone regeneration seems to be favorable to new bone formation. However, a comparative study to determine the optimal ratio of TiO_2_/HA as a bone material and UV irradiation time is needed.

## Figures and Tables

**Table 1:** Histomorphometry. Mean ± standard deviation values for new bone (NB) and the residual bone material (RBM) as percentages within surgically created defects (*n* = 12).

		Negative Control	Porous HA	TiO_2_-Coated HA	TiO_2_-Coated HA with UV
2 weeks	% NB	6.36 ± 1.72	18.78 ± 4.60 ^a^	28.63 ± 2.75 ^a, b^	36.32 ± 6.87 ^a, b, c^
	% RBM		10.56 ± 1.62	10.90 ± 1.78	14.53 ± 2.15 ^b^
8 weeks	% NB	13.90 ± 3.68	29.42 ± 5.81 ^a^	39.68 ± 6.70 ^a, b^	49.46 ± 6.54 ^a, b, c^
	% RBM		12.14 ± 5.22	14.64 ± 2.82	13.38 ± 3.44

NB: new bone, RBM: residual bone material. ^a^ Statistically significant difference compared to the negative control group (*p* < 0.05). ^b^ Statistically significant difference compared to the HA group (*p* < 0.05). ^c^ Statistically significant difference compared to the TiO_2_-coated HA group (*p* < 0.05).
